# Comparison of the most common isolates of postoperative endophthalmitis in South Korea; *Enterococcus* species vs coagulase-negative staphylococci

**DOI:** 10.1186/s12879-016-2038-5

**Published:** 2016-11-25

**Authors:** Ki Yup Nam, Hyun Wong Kim, Woo Jin Jeung, Jung Min Park, Jong Moon Park, In Young Chung, Yong Seop Han, Bu Sup Oum, Ji Eun Lee, Ik Soo Byon, Il Han Yun, Joo Eun Lee, Hee Sung Yoon, Dong Park, Byeng Chul Yu, Sang Joon Lee

**Affiliations:** 1 Department of Ophthalmology, College of Medicine, Kosin University, 262 Gamchun-ro, Seo-gu, Busan, South Korea; 2 Department of Ophthalmology, College of Medicine, Inje University, Busan, South Korea; 3 Department of Ophthalmology, College of Medicine, Dong-A University, Busan, South Korea; 4 Department of Ophthalmology, Maryknoll Hospital, Busan, South Korea; 5 Department of Ophthalmology, Graduate School of Medicine, Gyeongsang National University, Jinju, South Korea; 6 GM St. Mary Eye Clinic, Busan, South Korea; 7 Department of Ophthalmology, Graduate School of Medicine, Busan National University, Busan, South Korea; 8 Nunevit Eye Clinic, Busan, South Korea; 9 Sungmo Eye Hospital, Busan, South Korea; 10 Crystal Eye Clinic, Busan, South Korea; 11 Department of Preventive Medicine, College of Medicine, Kosin University, Busan, South Korea; 12 Institute for medicine, College of Medicine, Kosin University, Busan, South Korea

**Keywords:** Endophthalmitis, *Enterococcus faecalis*, *Staphylococcus epidermidis*, Coagulase negative staphylococci, Postoperative endophthalmitis, Posttraumatic endophthalmitis, Endogenous endophthalmitis

## Abstract

**Background:**

To compare the related factors or manifestations of the two most common isolates of post-operative endophthalmitis, which were *Enterococcus* spp. and coagulase-negative staphylococci (CNS) in South Korea.

**Methods:**

Medical records were reviewed for cases of post-operative endophthalmitis caused by *Enterococcus* spp. and CNS at eight institutions between January 2004 and July 2010. Various factors including age, sex, residence, systemic diseases, smoking and drinking history, and best corrected visual acuity, and length of time between causative intraocular surgery and symptom development were compared between the two groups.

**Results:**

The total number of post-operative endophthalmitis cases was 128 and in 116 cases, microbiological culture tests from the aqueous humor or vitreous were performed. Among these cases, 67 (57.8%) were culture proven. Among these 67 cases, 19 (28.4%) were caused by *Enterococcus* spp., 14 (20.9%) were caused by *Staphylococcus epidermidis* endophthalmitis, and 5 (7.5%) were caused by other CNS spp. Age, sex, causative procedure, past medical history, social history, and laterality were not different in the two groups. Mean initial and final visual acuity were significantly worse in the *Enterococcus* spp. endophthalmitis group than in the CNS group (*p* = 0.049, 0.042, respectively). Length of time between the causative procedure and symptom development was significantly shorter in cases of *Enterococcus* spp. endophthalmitis (*p* = 0.004).

**Conclusions:**

*Enterococcus* spp. induce more severe and rapid-onset postoperative endophthalmitis than CNS. Infectious endophthalmitis developed within 2 days after cataract operation could be caused by Enterococcus spp. and have chance to be poor prognosis in South Korea.

## Background

The type of microorganism involved in a case of infectious endophthalmitis is one of the most important factors determining visual prognosis [1–3]. Bacteria are the most common causative microorganisms although the reported incidences of bacterial strains vary. The most frequent causative strain in postoperative endophthalmitis was thought to be coagulase-negative staphylococci (CNS) including *Staphylococcus epidermidis* [4–7]. However, *Enterococcus* species (spp.) have recently emerged as a leading cause of endophthalmitis in South Korea and Sweden. The authors found that *Enterococcus faecalis* was the most common causative organism, accounting for 28.4% of all culture positive post-operative cases of endophthalmitis [1]. Additionally, a prospective study by Friling et al. on Swedish patients found that *Enterococcus* spp were the leading cause of post-operative endophthalmitis [8]. Interestingly, their previous study performed between 2002 and 2004 showed that *Enterococcus* spp. were the second most common isolate [9]. In their the next paper covering 2005 ~ 2010, *Enterococcus* spp. had become the most common microorganism [8]. The increased incidence of *Enterococcus* spp. was associated with worse visual outcomes of Kim and Friling’s study compared to previous studies in which CNS were the most common isolates including the endophthalmitis vitrectomy study (EVS) [4, 7, 10]. The emergence of *Enterococcus* spp. as isolate in infectious endophthalmitis warrants investigation of the comparative clinical features, visual outcome, and antibiotics susceptibilities between isolated *Enterococcus* spp. and CNS in South Korea. However, there have been no comparisons of the two isolates cultured from infectious endophthalmitis in a single study.

EVS is a prospective randomized study on postoperative endophthalmitis in the USA. In the EVS, *Enterococcus* spp. are only 7 cases (2.2%) among 323 isolates. Coagulase-negative micrococci including *S. epidermidis* comprise the majority of cases at 226 (70.0%). The number of *Enterococcus* spp. was too small to be directly compared to CNS, so the *Enterococcus* spp. were included in the “other gram positive group” and was analyzed with other groups in the EVS. The clinical features of the infection caused by the two major causative isolates, CNS and *Enterococcus* spp. were not available in the EVS, similar to other previous endophthalmitis studies.

*Enterococcus* spp. are gram-positive cocci in chains and leading causes of nosocomial infections and subacute endocarditis [11]. The most common pathogenic strain among *Enterococcus* spp is *E. faecalis* which is a natural inhabitant of the mammalian gastrointestinal tract and is found in soil, sewage, water, and food frequently through faecal contamination. It is also found in the normal conjunctival flora at a low percentage and endophthalmitis caused by *Enterococcus* spp. has a very unfavorable prognosis for visual acuity [5, 12]. There have been several articles about *Enterococcus* spp. analyzing antibiotic sensitivities and visual outcomes of *E. faecalis* endophthalmitis. Those studies included multiple categories of endophthalmitis such as postoperative, traumatic, and endogenous endophthalmitis over a 10 year study period [13–15]. The incidence of *E. faecalis* endophthalmitis has been historically been low, such that a single comparison between *E. faecalis* endophthalmitis and endophthalmitis due to other microorganisms has not been published until now. Therefore, it should be worth to compare clinical features of postoperative endophthalmitis caused by *E. faecalis* and CNS in a single study which have relatively short study period, perform in a same local area and under similar climate, and hygiene circumstances.

The purpose of the current paper was to analyze the clinical characteristics of *E. faecalis* and CNS in cases of postoperative endophthalmitis to differentiate between causative organisms and to inform a patient’s prognosis in terms of clinical course and visual outcome based on the data collected from consecutive 5-years study period.

## Methods

This study analyzed the medical records of 197 eyes of 197 patients who were diagnosed and treated with infectious endophthalmitis in 7 medical institutions in Busan, Gyeongsangnam-do from January, 2004 to July, 2010. We used data from same patients pool with our previous study [1]. Institutional review board approval was obtained from Gyeongsang National University and the protocol of this study adhered to the provisions of the Declaration of Helsinki. We obtained the approval of patients for use of their clinical data with written informed consent.

Endophthalmitis was diagnosed based on the clinical manifestations of patients, and confirmed through a culture when patients were suspected of having endophthalmitis. Cultures were done by aspiration of the aqueous humor or vitreous.

Several factors and clinical manifestations were compared between the 2 groups of post-operative infectious endophthalmitis caused by *Enterococcus* spp. and CNS. Patient medical records were checked for demographic variables including age, sex, residential area, the presence of diabetes mellitus (DM) and hypertension (HTN), drinking and smoking status, visual acuity at the time of initial diagnosis, causative intraocular surgery of endophthalmitis, onset of symptom, follow-up visual acuity, treatment method and complications of endophthalmitis. Initial treatment methods were simply classified into vitrectomy (with or without intravitreal antibiotics injection) and intravitreal antibiotics injection (with or without delayed vitrectomy). Antibiotic agents, which were injected intravitreally at the end of vitrectomy or used systemically were investigated. In terms of residence, patients living in cities were classified as living in an urban area, while patients living in districts smaller than cities were classified in the rural area group.

Best corrected visual acuity was converted to logMAR for statistical analysis, and counting fingers, hand motion, light perception, and non-light perception was substituted as 1.9, 2.3, 2.7, or 3.0, respectively [14]. Visual acuity measured at 2 months after endophthalmitis treatments was used as the final visual acuity. In regard to the onset of symptoms, the length of time from the causative procedure to postoperative symptom development was measured based on the onset of symptoms such as a decrease in visual acuity, congestion, and pain.

Statistical tests were conducted using SPSS version 18.0. Fisher’s exact test was performed to analyze sex, residence, the presence of DM and HTN, drinking and smoking status, causative intraocular surgery of endophthalmitis, treatment method and complications of endophthalmitis. The Mann–Whitney U test was performed to analyze age, initial and final visual acuity, and onset of symptoms.

## Results

Among a total of 197 patients diagnosed with and treated for endophthalmitis, post-operative endophthalmitis developed in 128 cases after intraocular surgery, and microbiological culture tests of the aqueous humor or vitreous was performed in 117 cases. Of these, microorganisms were identified in 67 eyes, and *Enterococcus* spp. accounted for 28.4% of all identified strains (19 eyes, *E. faecalis*: 17, *E. faecium*: 2), followed by *S. epidermidis* at 20.9% (14 eyes) and other CNS at 7.5% (5 eyes). In *Enterococcus* spp. group, 6 patients (31.6%) had aqueous culture only, 11 (57.9%) had vitreous culture only, and 2 (10.5%) had both. And in CNS group, 5 patients (26.3%) had aqueous culture only, 11 (57.9%) had vitreous culture only, and 3 (15.8%) had both.

Other cases of 29 eyes with culture positive results were 2 cases of *Staphylococcus aureus*, 1 case of *Staphyloccus warneri*, 9 cases of *Streptococcus* species, 3 cases of G (+) rods, 6 cases of *Pseudomonas species*, 6 cases of other G(−) rods, and 2 cases of fungi.

The characteristics of patients with *Enterococcus* spp. and CNS, respectively, the two most common causes of endophthalmitis, were compared. The mean ages of patients was 73.7 (±6.5) and 69.6 (±7.5) years for patients with *Enterococcus* spp. and CNS, respectively, showing no statistical difference. The male to female ratio was similar in the two groups. Causative surgery included a case of trabeculectomy in the *Enterococcus* spp. endophthalmitis group and two trabeculectomy cases in CNS endophthalmitis group, and the other causative operations were all cataract surgery (phacoemulsification and intraocular lens implantation). No significant differences were observed between factors including the presence of DM and HTN, or smoking and drinking history (Table 1).

**Table 1:** Comparison of various related factors between CNS and *Enterococcus* spp. groups

	*Enterococcus* spp. group (*n* = 19)	CNS group (*n* = 19)	*p*
Age	69.6 (±7.5)	73.7 (±6.5)	0.09^a^
Sex (%, eyes)			1.00^b^
Male	36.8 (7)	31.6 (6)	
Female	63.2 (12)	68.4 (13)	
DM (%, eyes)	21.1 (4)	42.1 (8)	0.29^b^
HTN (%, eyes)	47.4 (9)	63.2 (12)	0.51^b^
Drinking (%, eyes)	26.3 (5)	21.1 (4)	1.00^b^
Smoking (%, eyes)	15.8 (3)	21.1 (4)	1.00^b^
Causative procedure (%, eyes)			1.00^b^
Phacoemusification	94.7 (18)	89.5 (17)	
Trabeculectomy	5.3 (1)	10.5 (2)	
Laterality (OD : OS)	12 : 7	12 : 7	1.00^b^
Residential area (%, eyes)			1.00^b^
Urban area	84.2 (16)	78.9 (15)	
Rural area	15.8 (3)	21.1 (4)	

^a^Mann Whitney U test

^b^Fisher exact test

For patients with *Enterococcus* spp. endophthalmitis, the percentages of those dwelling in an urban area or a rural area was 84.2% (16 patients) and 15.8% (3 patients), respectively. For patients with CNS endophthalmitis, the percentages of those dwelling in an urban area or a rural area was 78.9% (15 patients) and 21.1% (4 patients), respectively (*p* > 0.05) (Table 1).

**Fig. 1:**
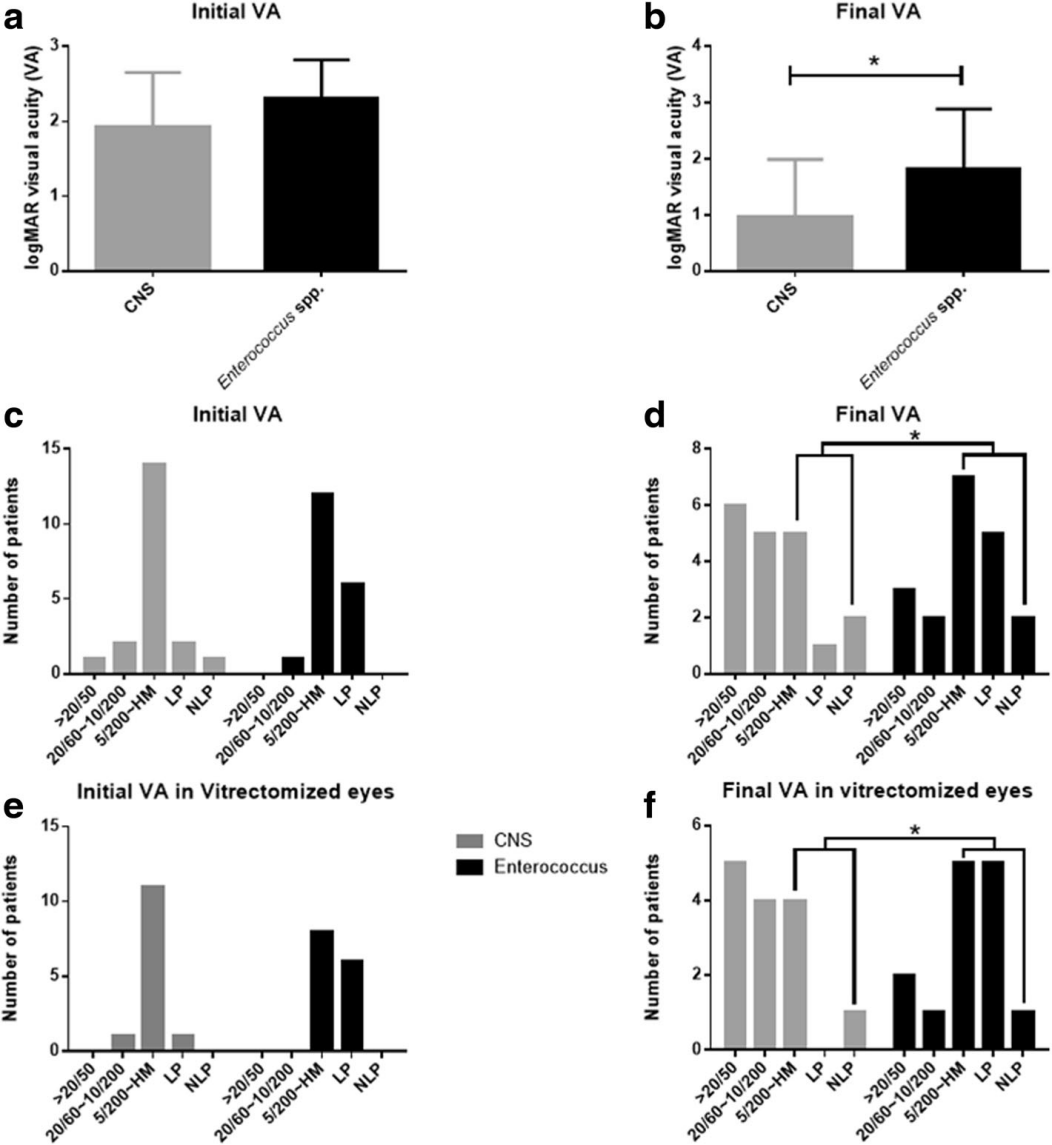
Comparison of mean initial and final visual acuity in CNS and *Enterococcus* spp. Groups. **a**, **b** The initial visual acuities showed no significant difference between CNS and *Enterococcus* spp. group, but the final visual acuity were significantly worse in *Enterococcus* spp. (*p* = 0.02, t-test). **c**, **d** The subgroups of initial and final visual acuities showed that the number of patients who were between 5/200 and NLP were significantly more in the *Enterococcus* spp. group (*p* = 0.049, Fisher’s exact test). **e**, **f** Vitrectomy as a initial treatment could not change the pattern of initial and final visual acuity of the *Enterococcus* spp. group

Mean initial visual acuity was 2.27 (±0.51) in the *Enterococcus* spp. endophthalmitis group and 1.92 (±0.72) in the CNS endophthalmitis group (*p* = 0.049). Mean final visual acuity was 1.73 (±1.06) in the *Enterococcus* spp. endophthalmitis group, and 0.78 (±1.02) in the CNS endophthalmitis group, which indicates a significantly worse visual outcome in the *Enterococcus* spp. group. (*p* = 0.042). The percentage of patients with a final visual acuity of less than 5/200 was 73.6% (14 eyes) in the *Enterococcus* spp. endophthalmitis group, and 36.8% (7 eyes) in the CNS endophthalmitis group (*p* = 0.048) (Fig. 1).

In both groups, 5 of 19 patients (26.3%) each were treated with intravitreal antibiotics injection as the initial treatment. In all cases, vancomycin and ceftazidime were used as intravitreal antibioitics for initial treatment. 4 of 5 patients (80.0%) in CNS group and 3 of 5 (60.0%) in *Enterococcus* spp. group received intravenous antibiotics including vancomycin, 3rd, 4th cephalosporin. 2 eyes in the *Enterococcus* spp. group received vitrectomy eventually. Thus, 15.8% (3 eyes) and 26.3% (5 eyes) of patients were treated with intravitreal antibiotics injection alone without undergoing vitrectomy in the *Enterococcus* spp. endophthalmitis group and CNS endophthalmitis group, respectively.

The final visual acuity of patients who received initial vitrectomy was analyzed and it was significantly worse in the *Enterococcus* spp. group (1.89 ± 1.0) than CNS group (0.79 ± 0.8) (*p* = 0.019). The percentage of initial vitrectomy group patients who had a final visual acuity less than 5/200 was 81.6% in the *Enterococcus* spp. group and 30.7% in the CNS group. There was no significant difference in initial and final visual acuity in the *Enterococcus* spp. endophthalmitis group; however, visual acuity significantly improved in the CNS group after pars plana vitrectomy (*p* = 0.016, Fig. 1).

Complications that developed after vitrectomy included corneal opacification, corneal edema, retinal detachment, and others, but no significance difference was shown between the two groups.

**Fig. 2:**
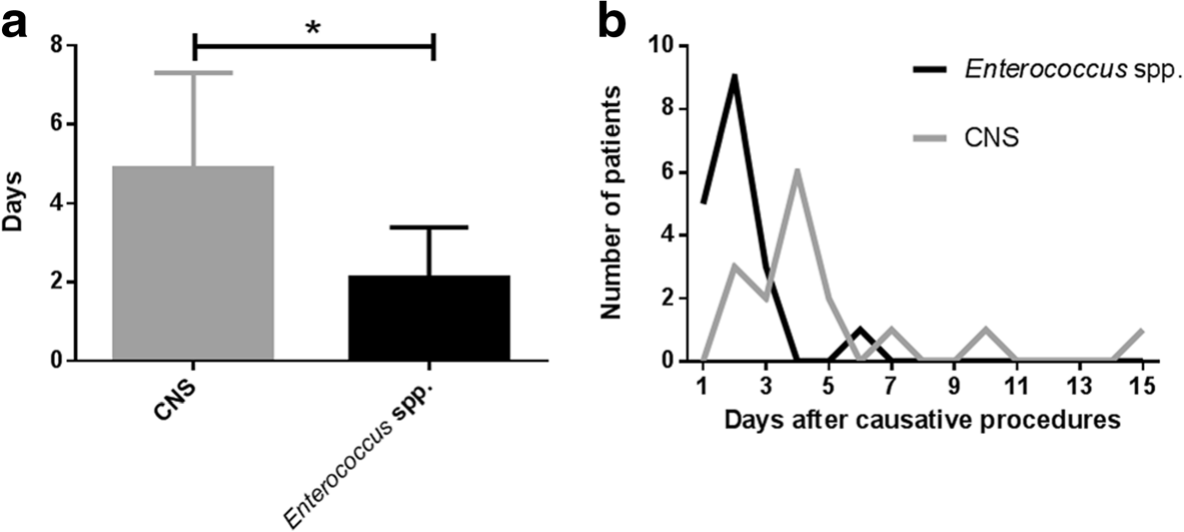
Duration from causative procedure to symptom development. **a** Mean duration from causative procedure to symptom development, **b** Distribution chart of symptom development duration (*p* < 0.05)

The average number of days from the causative operation to symptom development was 2.1 (±1.2) days in the *Enterococcus* species endophthalmitis group and 4.8 (±2.4) days in the CNS group than the other group (*p* < 0.001). The distribution chart for symptom onset showed a peak on day 2 in the group with *Enterococcal* endophthalmitis (Fig. 2).

## Discussion

Among the causative strains of post-operative infectious endophthalmitis, *Enterococcus* spp. have emerged as the most commonly detected strain cluster, followed by CNS including *S. epidermidis* in South Korea and Sweden [1, 8].

In our previous study, we found a interesting results about antibiotics susceptibility of cultured microorganisms. Fluoroquinolones showed poor activity against *E. faecalis*, but 100% of the isolates showed susceptability to vancomycin and imipenem and 92.9% showed susceptability to ampicillin [1]. And we suppose that the increase of *Enterococcus* spp. as the cause of post-operative endophthalmitis may be associated with intrinsic resistance to moxifloxacin, the 4^th^ generation fluoroquinolone, which is widely used nowadays.

In the present study, the endophthalmitis-related factors and clinical manifestations of these two strains were compared. No difference were found in the basic clinical characteristics such as age, sex, surgery type, the prevalence of DM and HTN, or smoking and drinking history. Since *Enterococcus* species is one of the major nosocomial pathogen transmitted through fecal contamination, it was assumed that there might be an association between place of residence and strain distribution when analyzing patients divided into urban and rural residence. However, no correlations were found for this variable.

The mean initial and final visual acuity was significantly worse in patients with *Enterococcus* spp. endophthalmitis than CNS (*p* = 0.049, *p* = 0.042 respectively), and there were also significantly more cases with a final visual acuity of less than 5/200 in the *Enterococcus* spp. group (*p* = 0.048). Among the several articles about *Enterococcus* spp., only Chen’s paper showed the *Enterococcus* spp. data for postoperative endophthalmitis, while the others included *Enterococcus* spp. cultured from all mixed endophthalmitis categories. Thus, it is difficult to compare the current results to previous *Enterococcus* spp. endophthalmitis studies. Nevertheless, regarding initial visual acuity, the percentage of patients with a visual acuity below light perception was 69.2% in the studies by Rishi et al. and Chen et al., which is significantly higher than the 31.6% (6/19 eyes) found in this study. The percentage of patients with a final visual acuity below light perception was 69.0% in the study of Chen et al., also significantly higher than the 36.8% (7/19 eyes) found in the current study [13, 14]. Visual acuity at the time of the initial and final diagnosis was more favorable in this study. This difference may be due to shorter duration of time between the causative procedure and diagnosis and treatments in this study compared to previous studies.

Intravitreal antibiotic injection alone was performed less frequently in the *Enterococcus* spp. endophthalmitis group, and vitrectomy was performed more frequently in the *Enterococcus* spp. endophthalmitis group. This could reflect the fact that the clinical course of *Enterococcus* spp. endophthalmitis was both more severe and progressed more rapidly. Thus, the retina surgeons might choose vitrectomy in *Enterococcus* spp. endophthalmitis as an initial treatment.

According to the visual outcome subanalysis in vitrectomized endophthalmitis cases, the *Enterococcus* spp. group showed a worse visual prognosis than the CNS group even though pars plana vitrectomy was performed in a greater proportion of patients. Moreover, the *Enterococcus* spp. group exhibited no significant difference between initial and final visual acuity after vitrectomy unlike the CNS endophthalmitis group. These findings indicate the need for further research on treatment strategies of *Enterococcus* spp. endophthalmtis.

Length of time between causative procedure and symptom development was significantly shorter in the *Enterococcus* spp. endophthalmitis group than the CNS group. The average number of days was 2.1 days in the *Enterococcus* spp. endophthalmitis group and 4.9 days in the CNS group. The distribution of symptom development showed a single peak at the second day in *Enterococcus*, and a peak at 4.3 days in CNS. Considering that these two isolates, *Enterococcus* spp. and CNS, represented the majority of isolates (56.8%) in these cases of postoperative endophthalmitis, the time of symptom onset after causative operation could be an important clue to help identify the causative organism.

This study has several limitations. First of all, the design of this study is retrospective. The increase of *Enterococcus* spp. endophthalmits may associated with the prophylactic antibiotics used for cataract surgery during pre- or peri-, post-operation period. And the differences of antibiotics according to the regions or countries could lead the increase of *Enterococcus* spp. as the causative strain of postoperative endophthalmits in South Korea and Sweden. However, we couldn’t collect the prophylactic antibiotics data enough to analyze. And secondly, the number of patients was too small, thus larger group study would be needed to support the results of our current study.

## Conclusion

*Enterococcus* spp. have recently been identified as a common cause of post-operative infectious endophthalmitis and are associated with a worse final visual acuity and earlier symptom development than CNS endophthalmitis. These findings could help clinicians differentiate between the two most common isolates of postoperative infectious endophthalmitis.

## Abbreviations

CNS: Coagulase-negative staphylococci
DM: Diabetes mellitus
*E. faecalis*: *Enterococcus faecalis*
*E. faecium*: *Enterococcus faecium*
EVS: Endophthalmitis vitrectomy study
HTN: Hypertension
*S. epidermidis*: *Staphylococcus epidermidis*
species: spp.
